# Urinary Prostaglandin E_2_ Metabolite and Pancreatic Cancer Risk: Case-Control Study in Urban Shanghai

**DOI:** 10.1371/journal.pone.0118004

**Published:** 2015-02-13

**Authors:** Jing Zhao, Jing Wang, Jinfeng Du, Hongli Xu, Wei Zhang, Quan-Xing Ni, Herbert Yu, Harvey A. Risch, Yu-Tang Gao, Ying Gao

**Affiliations:** 1 Key Laboratory of Nutrition and Metabolism, Institute for Nutritional Sciences, Shanghai Institutes for Biological Sciences, Chinese Academy of Sciences, University of Chinese Academy of Sciences, Shanghai, China; 2 Department of Epidemiology, Shanghai Cancer Institute, Renji Hospital, School of Medicine, Shanghai Jiaotong University, Shanghai, China; 3 Department of Pancreatic and Hepatobiliary Surgery, Fudan University Shanghai Cancer Center, Shanghai, China; 4 Epidemiology Program, University of Hawaii Cancer Center, Honolulu, HI, United States of America; 5 Department of Chronic Disease Epidemiology, Yale School of Public Health and Yale Cancer Center, New Haven, CT, United States of America; Spanish National Cancer Centre (CNIO), SPAIN

## Abstract

Pancreatic cancer has been increasing in importance in Shanghai over the last four decades. The etiology of the disease is still unclear. Evidence suggests that the COX-2 pathway, an important component of inflammation, may be involved in the disease. We aimed to evaluate the association between urinary prostaglandin E_2_ metabolite (PGE-M) level and risk of pancreatic cancer. From a recent population-based case-control study in Shanghai, 200 pancreatic ductal adenocarcinoma cases and 200 gender- and age- frequency matched controls were selected for the present analysis. Urinary PGE-M was measured with a liquid chromatography/mass spectrometric assay. Adjusted unconditional logistic regression was used to estimate odds ratios (ORs) and 95% confidence intervals (CIs). A positive association was observed between PGE-M leve and pancreatic cancer risk: OR = 1.63 (95% CI 1.01–2.63) for the third tertile compared to the first. Though the interactions were not statistically significant, the associations tended to be stronger among subjects with diabetes history (OR = 3.32; 95% CI 1.20–9.19) and higher meat intake (OR = 2.12; 95% CI 1.10–4.06). The result suggests that higher urinary PGE-M level may be associated with increased risk of pancreatic ductal adenocarcinoma.

## Introduction

Pancreatic cancer is one of the most fatal cancers in the world [1]. In the past 40 years, the incidence of this cancer has been increasing rapidly in China. Among all Chinese cities, Shanghai has the highest mortality from this disease [2]. In 1973, the urban Shanghai pancreatic cancer annual incidence rates were 3.66 per 10^5^ for men and 3.20 per 10^5^ for women [3], whereas in 2000, the rates had substantially increased to 11.22 and 10.93, respectively [3]. The lack of effective techniques for early diagnosis or treatment leads to less than 3% 5-year survival [4]. Cigarette smoking, family history of pancreatic cancer, history of diabetes mellitus and ABO blood group have been linked to the disease, though these factors explain only a fraction of the disease etiology [5,6].

Inflammation has been hypothesized to play a role in carcinogenesis of the pancreas [7]. Epidemiologic studies have suggested that chronic pancreatitis may be involved in some cases of pancreatic cancer [8]. Cyclooxygenase-2 (COX-2), a major enzyme in inflammation, has shown increased protein expression in pancreatic cells during the multistep progression of pancreatic cancer [9] and increased mRNA level in pancreatic cancer compared to adjacent nontumor tissue [10,11]. Nonsteroidal anti-inflammatory drugs (NSAIDs) are inhibitors of COX enzymes and their tumor suppressor effects on pancreatic cancer have been observed in both vitro studies [10,12,13] and some epidemiologic studies [14,15]. During inflammation, COX-2 converts arachidonic acid to PGE_2_, which may promote tumor development through inhibition of apoptosis, decrease of cell-mediated immunity, or stimulation of angiogenesis [16,17].

Since pancreatic tissue is difficult to access directly, the development of non-invasive technologies, including biomarkers in peripheral blood, pancreatic juice, or urine, could facilitate early detection of the disease [6]. Prostaglandin E_2_ metabolite (PGE-M) is the urinary metabolite of PGE_2_ and it can be used as an index of systemic PGE_2_ production_._ Several studies have observed that high levels of urinary PGE-M have been associated with increased risk of cancers of the colon and rectum [18–20], stomach [21] and breast [22,23], suggesting that urinary PGE-M might be associated with other inflammation-related cancers, including pancreatic cancer. Our study aimed to explore the association between urinary PGE-M levels and pancreatic cancer risk in a case-control study conducted in urban Shanghai.

## Materials and Methods

### Study population

The current study was conducted within an existing case-control study of pancreatic cancer that has been described previously [24,25]. Briefly, the parent case-control study was performed from December 2006 to January 2011 in urban Shanghai. The subjects recruited were Shanghai residents aged between 35 and 79 years. An “instant case reporting system” was used to identify cases in 37 major hospitals. In total, 1241 patients newly diagnosed with pancreatic cancer were reported to the Shanghai Cancer Institute. Of these patients, 149 (12%) were unable to be contacted or refused to participate, and 184 (14%) were excluded because of diagnoses of benign tumors or non-pancreatic primaries, which left 908 confirmed pancreatic cancer patients in the study. Among them, 311 cases were histologically confirmed as pancreatic ductal adenocarcinoma according to WHO classification of Tumors of the Digestive System. For controls, 1,653 candidates randomly selected from Shanghai Residents Registry were contacted. Among those, 586 (35%) were excluded for other malignant diseases (94), deceasing (30) and refusal (462) and there were 1067 candidates recruited as controls. All participants were interviewed in-person to collect information on cigarette smoking, family history of cancer, personal medical conditions, dietary intakes and various other factors. Body mass index (BMI, weight/height^2^) was calculated from reported height at age 21 and body weight of a year before interview. Participants were asked to retain overnight urine from 8:00pm to the next 8:00am and the urine was collected before any treatment. The 12-hour urine samples were collected into sterile cups containing 1g ascorbic acid and 5mg EDTA. Samples were kept on ice (4°C) for transportation to the laboratory and processed within 1 hour into long-term storage at −80°C.

For the current analysis, 200 ductal adenocarcinoma cases with pathology diagnoses were randomly selected from the parent case-control study. Two hundred parent-study controls were randomly frequency matched to the 200 cases on gender and age. This study was approved by the institutional human subjects review boards of the Shanghai Cancer Institute and Yale University, and all of the participants provided written informed consent.

### PGE-M measurement

Liquid chromatography/mass spectrometric assay (LC/MS/MS) was used to measure levels of urinary 11-α-hydroxy-9,15-dioxo-2,3,4,5-tetranor-prostane-1,20-dioicacid (PGE-M), the major metabolite of PGE_2_. The method has been described previously [26]. Briefly, 0.5mL urine with 15.0 μL (200ng/mL) of deuterated internal standard (PGE-M-d6) was adjusted to pH 3 with HCl, and PGE-M in the sample was converted to its *O*-methyloxime derivative with methyloxime HCl. After the urine samples were incubated in a 37°C water bath for 30 min, SepPak plus-C18 was applied to extract the methyloximed PGE-M using ethyl acetate as eluent. The eluate was then evaporated to dryness and residues were redissolved in 80.0μL of reconstituted mobile phase solution. Analysis was performed by liquid chromatography (Phenomenex Kinetex-C18 column) attached to an MDS Sciex API-4000 mass spectrometer with ESI probe. For endogenous PGE-M, the predominant product ion, m/z 336 representing [M-(OCH_3_+H_2_O)], and the analogous ion, m/z 339 [M-(OC[^2^H_3_]+H_2_O)] for the deuterated internal standard, were monitored in selected reaction monitoring (SRM) mode. Quantification of endogenous PGE-M used the ratio of the mass chromatogram peak areas of the m/z 336 and 339 ions. Urinary creatinine levels were measured using an Olympus AU5400 clinical chemistry analyzer with 0.1ml urine samples. The 400 samples were tested in five batches. Forty quality-control (QC) specimens (10%) from a single sample were randomly dispersed among the test-sample batches. The laboratory technician was blinded to case/control and QC sample status. The coefficients of variation for PGE-M and creatinine of the QC samples were 4.3% and 3.7%, respectively. The CV for the QC sample PGE-M corrected by creatinine was 4.8%. One sample’s result was below the lower limit of measurement (0.2ng/ml) and was assigned to the lowest PGE-M level group for the analysis.

### Statistical Analyses

Student’s t-test for continuous variables and the chi-square test for categorical variables were used to examine differences in basic characteristics between cases and controls. Urinary PGE-M levels were standardized by urinary creatinine values and were expressed as ng/mg creatinine. As the distribution of PGE-M was right-skewed, values were log_10_ transformed for analysis of continuous trends. Subjects were classified into tertiles according to the PGE-M distribution in controls, with lowest tertile as the reference group. We used two methods to choose the covariates included in the multivariable-adjusted models: (1) the potential confounder is associated with pancreatic cancer risk and with the PGE-M levels and the potential confounder changes the risk estimate by at least 10%; (2) the *P* value for -2Loglikelihood test is less than 0.05. In the final models, we use two different sets of confounders. Based on the former method, we used the basic model included gender and age as confounders. According to the second method, except for gender and age, attained education, family history of pancreatic cancer, and diabetes history were involved in the full model. To prevent the reverse causality, diabetes history was divided into three categories: no diabetes history, self-reported diabetes diagnosed less than 3 years before interview and diagnosed at least 3 years before interview.

To explore combined effects and modification effects of some *a priori* risk factors on the association between PGE-M and pancreatic cancer risk, stratified analyses were conducted by gender, history of diabetes mellitus, meat intake, vegetables/fruits intake and current aspirin usage et al. Continuous variables were classified into high *vs* low levels based on the median levels in controls. Interactions were evaluated with the log likelihood ratio statistic. All analyses were conducted with SAS 9.3 software and all *P*-values are two-sided.

## Results

Basic characteristics of case and control subjects are given in Table 1. Cases and controls were similar in body mass index, smoking exposure, family history of cancer, pancreatitis, vegetables/fruits intake, dietary energy density, regular green tea drinking, and current usage of aspirin. Compared to controls, cases had higher levels of education and greater meat intakes, and were more likely to have family histories of pancreatic cancer, personal histories of diabetes and higher PGE-M levels. Among all the 200 cases, 177 (88.5%) subjects were in stage.

**Table 1:** Characteristics of selected pancreatic cancer cases and controls, case-control study in urban Shanghai.

Characteristics^a^		Controls(n = 200)	Cases(n = 200)	*P* ^b^
gender				1.00
	male	124(62.0)	124(62.0)	
	female	76(38.0)	76(38.0)	
Age(years)		64(57–72)	63(57–71)	0.88
Body mass index(kg/m^2^)		23.0(21.1–25.4)	23.6(21.8–25.4)	0.38
Education				*0.004*
	High school/lower	168(84.0)	144(72.0)	
	College/higher	32(16.0)	56(28.0)	
Smoke status				0.91
	never	104(52.0)	100(50.0)	
	ever	22(11.0)	22(11.0)	
	current	74(37.0)	78(39.0)	
Cigarette use(pack-year)				
	0	104(52.0)	99(49.5)	0.98
	0.025-<13.7	22(11.0)	22(11.0)	
	13.7-<27.4	26(13.0)	29(14.5)	
	27.4-<41.1	28(14.0)	31(15.5)	
	≥41.1	20(10.0)	19(9.5)	
Family history of cancer		68(34.0)	79(39.5)	0.25
Family history of pancreatic cancer		2(1.0)	12(6.0)	*0.0115* ^c^
Diabetes status		21(10.5)	49(24.5)	*0.0002*
Diabetes history				*0.0003*
	No	179(89.5)	151(75.5)	
	Less than 3 years	4(2.0)	20(10.0)	
	More than or equal to 3 years	17(8.5)	29(14.5)	
Pancreatitis		1(0.5)	6(3.0)	0.06
Meat intake,g/d		102.5(76.0–136.2)	115.0(79.7–162.1)	*0.003*
Vegetables/fruits intake,g/d		534.8(385.1–676.8)	522.0(396.4–652.1)	0.22
Energy density,kJ/g		5.8(5.1–6.8)	5.9(5.1–6.8)	0.62
Regular green tea drinking		107(53.5)	113(56.5)	0.55
Current aspirin usage		20(10.0)	18(9.0)	0.73
Pancreatic cancer stages				
Stage Ⅰ			12(6.0)	
Stage Ⅱ			177(88.5)	
Stage Ⅲ			11(5.5)	
PGE-M(ng/ml)		7.85(5.00–12.80)	8.34(4.35–13.10)	
Creatinine(mg/ml)		0.67(0.43–0.97)	0.57(0.41–0.82)	
Standardized PGE-M (ng/mg Cr)		11.45(8.94–15.37)	13.48(9.01–20.55)	*0.025* ^d^

^a^Continuous variables were expressed as median (interquartile) and categorical variables were expressed as frequency(percentage among cases or controls).

^b^ *P*-value were calculated from student’s t test for continuous variables and χ^2^-test for categorical variables.

^c^ *P*-value were calculated from Fisher’s exact test.

^d^ *P*-value were calculated from student’s t test for log_10_ transformed standardized PGE-M.

In the basic model, compared to the lowest tertile, the highest level of urinary PGE-M was associated with increased risk of pancreatic cancer, with odds ratio (OR) estimate of 1.63 (95% CI 1.01–2.63) (Table 2). With regard to the full model, attained education, family history of pancreatic cancer, and diabetes history were not found to alter the magnitudes of association for PGE-M (Table 2).

**Table 2:** Association of urinary PGE-M levels and risk of pancreatic cancer.

	T1	T2	T3
PGE-M range (ng/mg Cr)	3.26–9.63	9.67–13.66	13.72–183.25
PGE-M Median (ng/mg Cr)	7.89	11.39	16.79
No. of controls	66	66	68
No. of cases	59	43	98
Basic model ORs(95% CI)^a^	1	0.74(0.43–1.25)	*1.63(1.01–2.63)*
Full model ORs (95% CI)^b^	1	0.76(0.44–1.32)	*1.66(1.01–2.73)*

Abbreviation: OR, odds ratio; CI, confidence interval; Cr, creatinine.

^a^basic model: adjusted for gender and age.

^b^full model: adjusted for gender, age, education levels, family history of pancreatic cancer, and diabetes history (three categories: no diabetes history, diagnosed less than 3 years before interview, diagnosed at least 3 years before interview).

**Fig 1:**
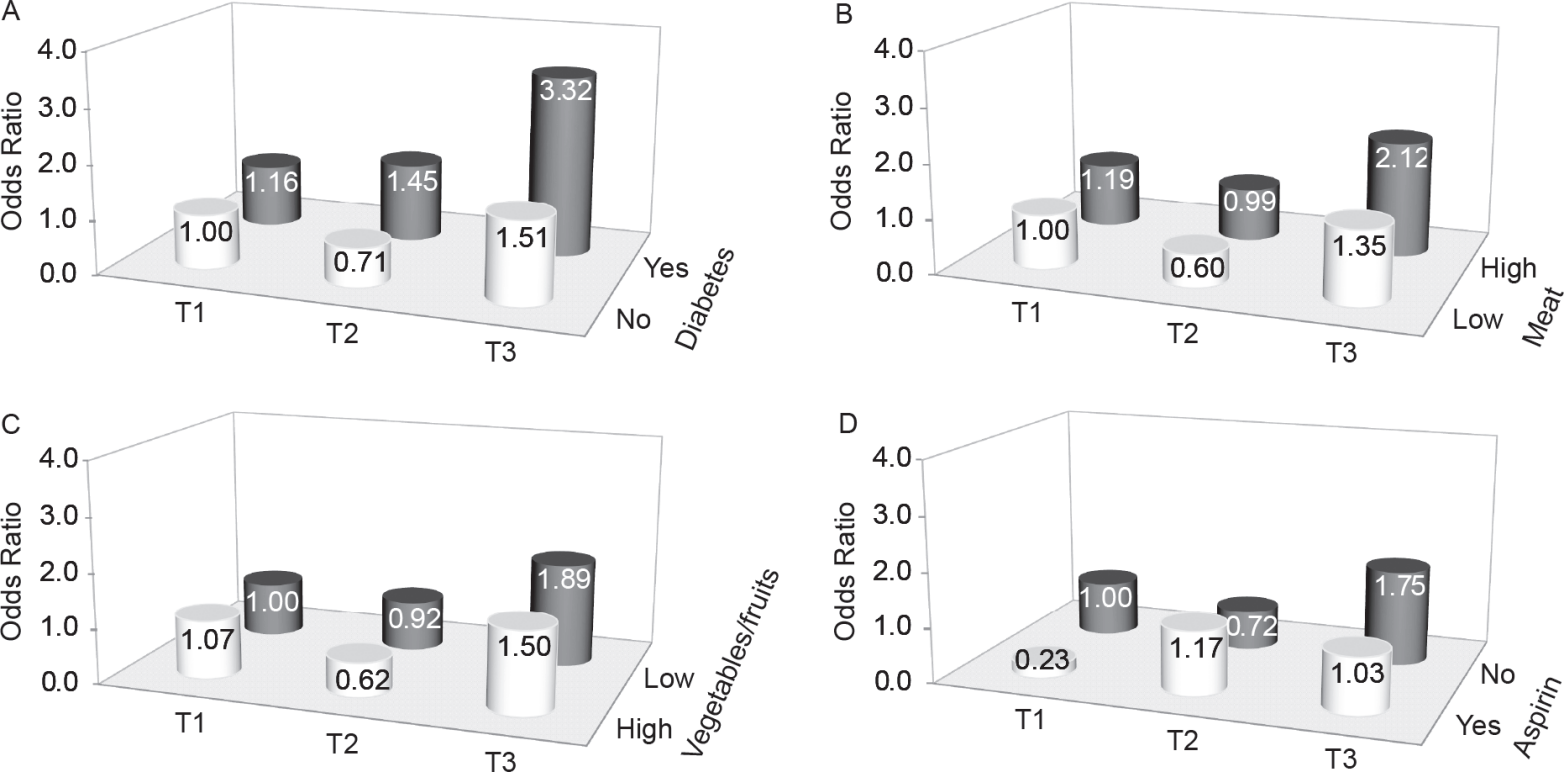
Combined effects of PGE-M and some a priori factors on risk for pancreatic cancer. Adjusted ORs for pancreactic cancer according to the tertiles of PGE-M and diabetes status (A), meat intake (B), vegetables/fruits intake (C), and current aspirin usage (D). Adjusted for gender and age. Diabetes History was considered positive for self-reported diabetes diagnosed at least 3 years before interview.

We explored the combined effects of PGE-M and several a priori risk factors on pancreatic cancer risk, including gender, diatetes status (yes and no), dietary intake of meat and fruits/vegetables (high and low), and current aspirin usage (yes and no) (Fig. 1, S1 Table). The positive association between PGE-M and pancreatic cancer risk tended to be stronger among participants who had diabetes history and higher meat intake with ORs of 3.32 (95% CI 1.20–9.19) and 2.12 (95% CI 1.10–4.06), respectively (Fig. 1, S1 Table). We also explored potential modifying effects of these factors (S2 Table). The positive association for tertile 3 among all subjects was still significant among participants who reported not using aspirin: OR 1.69 (95% CI 1.02–2.80). Significant interactions of urinary PGE-M were not seen for the five factors (S2 Table). In addition, we explored the associations between urinary PGE-M levels and risk of pancreatic cancer by stages (S3 Table), and the association kept similar for stage II (OR = 1.76, 95% CI = 1.07–2.89).

## Discussion

In the current case-control study, we observed a positive association between high urinary PGE-M level and increased risk of pancreatic cancer. The positive association with high urinary PGE-M level was still significant after exclusion of individuals with current aspirin usage and was stronger among subjects with diabetes history or higher meat intake.

PGE-M is the urinary metabolite of PGE_2_, and thus indirectly reflects blood levels of PGE_2_. Excreted urinary metabolites measured by LC/MS/MS provide highly accurate indicators of endogenous eicosanoid production in humans [26,27]. Oral administration of both non-selective and selective inhibitors of COX-2 can result in lower urinary PGE-M levels in healthy individuals, which suggests that urinary PGE-M can reflect COX-2 production and activity [26].

To our knowledge, six epidemiological studies to-date have explored associations between urinary PGE-M levels and cancer risk, including four studies of gastrointestinal neoplasms [18–21] and two studies of breast cancer [22,23]. Both a study on advanced/multiple colorectal adenoma [19] and a study on colorectal cancer [18] observed significant associations between PGE-M and disease risk. In a case-series study, urinary PGE-M levels among patients with Crohn’s disease, colorectal cancer and large adenomas were about two fold increased compared to patients who had small polyps or no polyps [20]. For gastric cancer [21], a significantly increasing trend in risk was observed with increasing PGE-M levels among women who had been diagnosed within 46 months after baseline urine collection. For breast cancer, the risk was increased with urinary PGE-M levels among postmenopausal women with no NSAIDs usage [22] or with a BMI<25 kg/m^2^ [23]. Consistent with these studies, we observed a positive association between high urinary PGE-M level and increased risk of pancreatic cancer. These results suggest that PGE-M, possibly as a marker of inflammation, could be associated with gastrointestinal cancer development.

PGE_2_ is one of the products of arachidonic acid catalyzed by COX-2, which has been suggested to play a role in the development of certain cancers [16,28]. COX-2 involvement may promote cancer cell proliferation, migration, invasion, and inhibit cell apoptosis via a number of signaling pathways [29]. PGE_2_ could also regulate angiogenesis and tumor immune-suppression in the tumor microenvironment [16]. Several clinical studies have observed that COX-2 is up-regulated in pancreatic adenocarcinoma [9–11]. COX-2 mRNA expression was more than 60-fold increased in pancreatic cancer tissue compared to adjacent non-tumor tissue [30]. In the hamster model of chemically induced ductal pancreatic adenocarcinoma, the selective COX-2 inhibitor, Celebrex, was observed to inhibit tumor growth in liver metastases [31]. Various epidemiological studies have suggested that inhibitors of COX-2 could protect against pancreatic cancer [32]. Furthermore, expression loss of 15-hydroxyprostaglandindehydrogenase (15-PGDH), an important enzyme involved in PGE_2_ degradation, has been linked to tumor formation, including colorectal cancer, lung cancer and transitional bladder cancer [29]. Thus, increased PGE_2_ may be associated with increased cancer risk, including pancreatic cancer.

Urinary PGE-M is subject to the influence of many factors. Aspirin, a COX-2 inhibitor is related to decreased PGE-M levels [26]. Interestingly, only 9.5% of individuals in our study took aspirin regularly; excluding the individuals with current aspirin usage strengthened the positive association between PGE-M and pancreatic cancer risk. This is adding further evidence supporting the negative relationship between aspirin and PGE-M. Smoking status has been observed to be positively associated with urinary PGE-M level smoking status, which had a dose-response effect [33]. Our data also suggested that urinary PGE-M was higher in ever smokers than never smokers (median (interquartile): 12.71(9.07–19.24) *vs* 11.51(8.77–16.45) ng/mg Cr). Increased BMI is associated with increased urinary PGE-M level [34]. Consistently, in our study, subjects with BMI more than 28kg/m^2^ had higher PGE-M level than subjects with BMI less than 24kg/m^2^ (median (interquartile): 13.55(10.76–18.41) *vs* 11.42(8.56–16.93) ng/mg Cr).

We also explored combined effects of several a priori factors with PGE-M on pancreatic cancer risk, and found a stronger association among subjects with higher meat intake. Meat contains abundant arachidonic acid [35] (the precusor of PGE_2_) and increased amount of dietary arachidonic acid probably augmented PGE_2_ formation [36, 37]. Therefore, meat intake here might be associated with pancreatic cancer through PGE_2_, which warrants further research. We also observed a significant association between PGE-M and pancreatic cancer among individuals with diabetes, which was reasonable since diabetes was an important risk factor related to pancreatic cancer [38].

Our study has several strengths. The diagnoses of all cases were validated by a panel of five clinical experts who used pathology reports, pathology slides and/or imaging materials, which minimized misclassification of cancer cases. Moreover, only ductal pancreatic adenocarcinoma patients were involved in our study, which increased the homogeneity of the disease studied.

A few potential limitations of the present study should also be considered. Firstly, as a case-control study, our analysis may be subject to reverse causality, where the presence of pancreatic tumors could theoretically increase serum levels of PGE_2_. However, we did not observe appreciable variation in urinary PGE-M according to disease stage. Secondly, the stratified analyses by stage were limited by power since 88.5% of cases were in stage II. Further study with larger sample size is needed. Thirdly, aspirin or NSAID usage at the time of interview may affect urinary PGE-M levels. However, there were only 18 cases and 20 controls reported current aspirin usage in the current study, and the association was still statistically significant after excluding these individuals.

In summary, the current study suggests that higher levels of urinary PGE-M may be associated with increased pancreatic cancer risk. Large prospective studies would be useful for further exploration.

## Supporting Information

S1 Table Combined effects of PGE-M and some *a priori* factors on risk for pancreatic cancer. (DOCX)

S2 Table Association of urinary PGE-M levels and risk of pancreatic cancer by potential modifiers. (DOCX)

S3 Table Association of urinary PGE-M levels and risk of pancreatic cancer in different cancer stages. (DOCX)
