# Supplementary material for: Urinary Prostaglandin E2 Metabolite and Pancreatic Cancer Risk: Case-Control Study in Urban Shanghai
Source: PLoS One. 2015 Feb 13;10(2):e0118004. doi: 10.1371/journal.pone.0118004 (PMC4332509; doi:10.1371/journal.pone.0118004)
Supplement: S1 Table — (DOCX) [file pone.0118004.s001.docx]

**Table S1. Combined effects of PGE-M and some *a priori* factors on risk for pancreatic cancer^a^**

| Gender^b^ | Male | |  | Female | |
| --- | --- | --- | --- | --- | --- |
|  | *N*_control/case_ | OR |  | *N*_control/case_ | OR |
| T1 | 43/37 | 1 |  | 23/22 | 1.11(0.54-2.32) |
| T2 | 37/26 | 0.83(0.42-1.62) |  | 29/17 | 0.70(0.33-1.48) |
| T3 | 44/61 | 1.62(0.90-2.93) |  | 24/37 | 1.84(0.92-3.70) |
| Diabetes history | No | |  | Yes^c^ | |
|  | *N*_control/case_ | OR (95% CI) |  | *N*_control/case_ | OR (95% CI) |
| T1 | 59/52 | 1 |  | 7/7 | 1.16(0.38-3.57) |
| T2 | 62/38 | 0.71(0.41-1.25) |  | 4/5 | 1.45(0.37-5.73) |
| T3 | 62/81 | 1.51(0.91-2.52) |  | 6/17 | *3.32(1.20-9.19)* |
| Meat intake | Low | |  | High | |
|  | *N*_control/case_ | OR (95% CI) |  | *N*_control/case_ | OR (95% CI) |
| T1 | 34/28 | 1 |  | 32/31 | 1.19(0.59-2.41) |
| T2 | 34/17 | 0.60 (0.27-1.31) |  | 32/26 | 0.99(0.48-2.04) |
| T3 | 32/36 | 1.35 (0.67-2.73) |  | 36/62 | *2.12 (1.10-4.06)* |
| Vegetables/fruits intake | Low | |  | High | |
|  | *N*_control/case_ | OR (95% CI) |  | *N*_control/case_ | OR (95% CI) |
| T1 | 35/30 | 1 |  | 31/29 | 1.07(0.52-2.18) |
| T2 | 32/25 | 0.92 (0.45-1.88) |  | 34/18 | 0.62 (0.29-1.32) |
| T3 | 33/53 | 1.89 (0.98-3.66) |  | 35/45 | 1.50(0.78-2.91) |
| Current aspirin usage | No | |  | Yes | |
|  | *N*_control/case_ | OR(95% CI) |  | *N*_control/case_ | OR(95% CI) |
| T1 | 59/53 | 1 |  | 7/6 | 0.23(0.03-2.06) |
| T2 | 62/39 | 0.72(0.41-1.26) |  | 4/4 | 1.17(0.27-5.00) |
| T3 | 59/90 | *1.75(1.06-2.91)* |  | 9/8 | 1.03(0.37-2.88) |

^a^ORs adjusted for gender and age.

^b^ORs adjusted for age.

^c^Diabetes History was considered positive for self-reported diabetes diagnosed at least 3 years before interview.
