# Supplementary material for: Urinary Prostaglandin E2 Metabolite and Pancreatic Cancer Risk: Case-Control Study in Urban Shanghai
Source: PLoS One. 2015 Feb 13;10(2):e0118004. doi: 10.1371/journal.pone.0118004 (PMC4332509; doi:10.1371/journal.pone.0118004)
Supplement: S2 Table — (DOCX) [file pone.0118004.s002.docx]

**Table S2. Association of urinary PGE-M levels and risk of pancreatic cancer by potential modifers^a^**

| Gender^b^ | Male | |  | Female | |
| --- | --- | --- | --- | --- | --- |
|  | *N*_control/case_ | OR |  | *N*_control/case_ | OR |
| T1 | 43/37 | 1 |  | 23/22 | 1 |
| T2 | 37/26 | 0.82(0.42-1.61) |  | 29/17 | 0.63(0.27-1.50) |
| T3 | 44/61 | 1.62(0.90-2.92) |  | 24/37 | 1.68(0.74-3.83) |
| *P* interaciton |  | 0.83 |  |  |  |
| Diabetes history | No | |  | Yes^c^ | |
|  | *N*_control/case_ | OR (95% CI) |  | *N*_control/case_ | OR (95% CI) |
| T1 | 59/52 | 1 |  | 7/7 | 1 |
| T2 | 62/38 | 0.71(0.41-1.25) |  | 4/5 | 1.74(0.28-10.69) |
| T3 | 62/81 | 1.50(0.90-2.50) |  | 6/17 | 3.11(0.72-13.48) |
| *P* interaciton |  | 0.76 |  |  |  |
| Meat intake | Low | |  | High | |
|  | *N*_control/case_ | OR (95% CI) |  | *N*_control/case_ | OR (95% CI) |
| T1 | 34/28 | 1 |  | 32/30 | 1 |
| T2 | 34/17 | 0.59 (0.26-1.31) |  | 32/26 | 0.86(0.42-1.78) |
| T3 | 32/36 | 1.33 (0.65-2.73) |  | 36/62 | 1.85 (0.96-3.56) |
| *P* interaciton |  | 0.75 |  |  |  |
| Vegetables/fruits intake | Low | |  | High | |
|  | *N*_control/case_ | OR (95% CI) |  | *N*_control/case_ | OR (95% CI) |
| T1 | 35/30 | 1 |  | 31/28 | 1 |
| T2 | 32/25 | 0.88 (0.43-1.82) |  | 34/18 | 0.64 (0.29-1.42) |
| T3 | 33/53 | 1.80 (0.93-3.50) |  | 35/45 | 1.53(0.76-3.07) |
| *P* interaciton |  | 0.72 |  |  |  |
| Current aspirin usage | No | |  | Yes | |
|  | *N*_control/case_ | OR(95% CI) |  | *N*_control/case_ | OR(95% CI) |
| T1 | 59/53 | 1 |  | 7/6 | 1 |
| T2 | 62/39 | 0.69(0.40-1.20) |  | 4/4 | 2.10(0.29-15.39) |
| T3 | 59/90 | *1.69(1.02-2.80)* |  | 9/8 | 1.36(0.27-6.78) |
| *P* interaciton |  | 0.51 |  |  |  |

^a^ORs adjusted for gender and age.

^b^ORs adusted for age

^c^Diabetes History was considered positive for self-reported diabetes diagnosed at least 3 years before interview.
