# Supplementary material for: Urinary Prostaglandin E2 Metabolite and Pancreatic Cancer Risk: Case-Control Study in Urban Shanghai
Source: PLoS One. 2015 Feb 13;10(2):e0118004. doi: 10.1371/journal.pone.0118004 (PMC4332509; doi:10.1371/journal.pone.0118004)
Supplement: S3 Table — (DOCX) [file pone.0118004.s003.docx]

Table S3. Association of urinary PGE-M levels and risk of pancreatic cancer in different

cancer stages^a^

| Stage | I (200/12) | | Ⅱ(200/177) | | Ⅲ (200/11) | |
| --- | --- | --- | --- | --- | --- | --- |
|  | *N*_control/case_ | OR | *N*_control/case_ | OR | *N*_control/case_ | OR |
| T1 | 66/7 | 1 | 66/49 | 1 | 66/3 | 1 |
| T2 | 66/1 | 0.14(0.02-1.18) | 66/39 | 0.80(0.46-1.38) | 66/3 | 1.23(0.23-6.63) |
| T3 | 68/4 | 0.59(0.16-2.18) | 68/89 | *1.76(1.07-2.89)* | 68/5 | 2.02(0.44-9.25) |

^a^ORs adjusted for gender and age
